# Microarray Analysis Identifies Key Differentially Expressed Circular RNAs in Aged Mice With Postoperative Cognitive Dysfunction

**DOI:** 10.3389/fnagi.2021.716383

**Published:** 2021-08-16

**Authors:** Yu-Qing Wu, Qiang Liu, Hai-Bi Wang, Chen Chen, Hui Huang, Yi-Man Sun, Lin-Hui Ma, Jie Wan, Yin-Ying Sun, Hui-Hui Miao

**Affiliations:** ^1^Jiangsu Province Key Laboratory of Anesthesiology, Xuzhou Medical University, Xuzhou, China; ^2^Department of Anesthesiology, Beijing Shijitan Hospital, Capital Medical University, Beijing, China

**Keywords:** circRNAs, expression profile, postoperative cognitive dysfunction, miRNAs, ceRNA network

## Abstract

Postoperative cognitive dysfunction (POCD) is a common complication in elderly patients. Circular RNAs (circRNAs) may contribute to neurodegenerative diseases. However, the role of circRNAs in POCD in aged mice has not yet been reported. This study aimed to explore the potential circRNAs in a POCD model. First, a circRNA microarray was used to analyze the expression profiles. Differentially expressed circRNAs were validated using quantitative real-time polymerase chain reaction. A bioinformatics analysis was then used to construct a competing endogenous RNA (ceRNA) network. The database for annotation, visualization, and integrated discovery was used to perform Gene Ontology (GO) and Kyoto Encyclopedia of Genes and Genomes (KEGG) enrichment analysis of circRNA-related genes. Moreover, protein-protein interactions were analyzed to predict the circRNA-regulated hub genes using the STRING and molecular complex detection plug-in of Cytoscape. Microarray screen 124 predicted circRNAs in the POCD of aged mice. We found that the up/downregulated circRNAs were involved in multiple signaling pathways. Hub genes, including* Egfr* and *Prkacb*, were identified and may be regulated by ceRNA networks. These results suggest that circRNAs are dysexpressed in the hippocampus and may contribute to POCD in aged mice.

## Introduction

Postoperative cognitive dysfunction (POCD) causes significant harm to elderly patients (Deiner and Silverstein, 2009). It may prolong hospital stay, increase medical expenses, reduce patients’ daily living ability, and may even be associated with an increase in long-term mortality. In addition, it may exacerbate the decline in cognitive dysfunction and even increase the rate of dementia (Steinmetz et al., 2009). Age is considered one of the possible risk factors associated with the incidence of POCD, as older adults have reduced cognitive reserve in the fragile brain (Terrando et al., 2011). Aged people are expected to be the largest surgical population in the future. Researchers have suggested that neuroinflammation, neuronal apoptosis, mitochondrial dysfunction, and synaptic dysfunction may contribute to POCD progression (Miao et al., 2017, 2018, 2019; Chen et al., 2019; Qiu et al., 2020). However, the exact mechanisms of POCD remain unclear, and the biomarkers are also lacking (Vutskits and Xie, 2016). Therefore, an in-depth study of the molecular mechanism of POCD is of clinical significance.

Circular RNA (circRNA) is a newly discovered class of approximately 100 nucleotides (nt) in length non-coding RNAs (ncRNAs) as a closed-loop structure. Studies have suggested that circRNAs are potential candidates for clinical diagnostic biomarkers (Jeck and Sharpless, 2014) because they are more stable. A variety of functions and mechanisms have been discovered with circRNAs, such as acting as microRNA (miRNA) sponges, binding proteins, or deoxyribonucleic acid (DNA) sequences, thereby regulating gene expression (Hansen et al., 2013; Memczak et al., 2013). They are stable in tissue and developmental stages in different species (Szabo et al., 2015). New studies suggest that circRNAs may be involved in different physiological or pathological processes and play an important role (Cheng et al., 2020; Wang et al., 2020; Zhang et al., 2020). Evidence has also shown that circRNAs are involved in neurological diseases such as Alzheimer’s disease (AD; Zhou et al., 2018; Ma et al., 2019; Chu et al., 2020). However, the differently expressed circRNAs in the hippocampus of aged mice and the role of circRNA in POCD are largely unknown.

Genome analysis has been used to identify functional genes in POCD for several years (Li et al., 2015). For example, Liu et al. (2019) found that many miRNAs were differentially expressed between POCD and control-aged mice (Chen et al., 2019; Su et al., 2019). Furthermore, long non-coding RNAs (lncRNAs) were found to be differentially expressed in mRNAs and might contribute to POCD (Li et al., 2019). However, the role of circRNAs in POCD in aged mice remains unclear. Therefore, the current study aimed to illustrate the circRNA expression profile and the potential functions and mechanisms of POCD.

## Materials and Methods

### Animal Experiments

C57BL/6 male mice (weighing 30–35 g and aged 18 months) were purchased from Nanjing University’s Model Animal Research Center. Mice were housed under standardized conditions. Before the start of the study, all mice were acclimatized for at least 1 week. The animal protocol was approved by the Animal Care and Use Committee of Xuzhou Medical University.

### Animal Model of POCD

Mice were separated into POCD (*n* = 10) and control groups (*n* = 10). The surgical operation of intramedullary fixation for open tibial fractures was performed in accordance with previous studies and with modifications (Feng et al., 2017; Chen et al., 2019). For the anesthesia induction period, 3.0% isoflurane was used, and 1.5% isoflurane was used for the maintenance period, which was supported by using 100% oxygen. First, we made an incision on the lateral side of the tibia, in order to expose the bone. Then, a minor hole was drilled into the tibial trochanter, and an intramedullary fixation needle was inserted. All mice were returned to their home cages after recovery from anesthesia. To manage postoperative pain, 2% lidocaine solution and 1% tetracaine hydrochloride glue were used locally twice daily.

### Behavioral Tests

We used the open field test (OFT) to estimate motor activity 2 h ahead of the fear conditioning test (FCT) training. Mice were allowed free exploration in the OFT for 5 min. We used the system camera to track and record the mouse movement automatically, and selected the total distance to ascertain locomotor ability.

The test and training stages were included in the FCT. The mice were allowed to adapt to the chamber environment for 2 min, given six pairs stimulation, and then maintained for another 1 min. The cycle consisted of a conditional stimulus (70 dB tone, 20 s), a trace interval (contextual break, 25 s), and an unconditional stimulus (0.70 mA electrical footshock, 2 s). Every cycle was randomly divided into breaks of 45–60 s.

### RNA Extraction

The hippocampal tissue of mice was preserved at −80°C after behavioral experimentation on day 3 postoperatively. TRIzol reagent (Invitrogen, Carlsbad, CA, USA) was used to extract total RNA.

### Microarray Detection

Kangchen Biotechnology (Shanghai, China) Arraystar circRNA microarray was used for RNA sample analysis and the obtained samples were labeled by Arraystar’s standard protocols. We used Limma for the original raw data processing.

### Quantification With PCR

The cDNA samples were configured with the real-time PCR reaction system. 2X PCR Master Mix (Arraystar) was used. The primer sequences used for the predicted circRNAs are listed in Table 1.

**Table 1:** The forward and reverse primers for qPCR.

Gene	Primers	Sequence (5′ to 3′)
mmu_circRNA_28795	Forward primer	AAGGAGGATACTAGCAGGTTGGA
	Reverse primer	TGTAGTAGTAGAAGCGTGA
		TGTATTGG
mmu_circRNA_44122	Forward primer	GACACACAGACAGACCCATACTGT
	Reverse primer	CTTCTCTGCTCTGCAAGGTGG
mmu_circRNA_22058	Forward primer	AGGCAGCACTCAATGGACAAG
	Reverse primer	ACCAGTCATGTCGTTTTCCTTTAC
mmu_circRNA_44559	Forward primer	CAAAGGTGTTGTGAAGGAAGTC
	Reverse primer	TTTCTGTATCACACGTGCGTAA
mmu_circRNA_45921	Forward primer	ATGTGGGAGCGTGGAGATAA
	Reverse primer	CATCACGGTCAGCATTCTTG
mmu_circRNA_22673	Forward primer	TCAAGTCCTGCACAAAGACAAC
	Reverse primer	GCAAGGCTCCTAAATCAAAGTC
Egfr	Forward primer	GCCATCTGGGCCAAAGATACC
	Reverse primer	GTCTTCGCATGAATAGGCCAAT
Prkacb	Forward primer	ATGGTTATGGAATACGTCCCTGG
	Reverse primer	AATTAAGAGGTTTTCCGGCTTGA
β-actin	Forward primer	GTACCACCATGTACCCAGGC
	Reverse primer	AACGCAGCTCAGTAACAGTCC

### Annotation and Functional Prediction of Up/Downregulated circRNAs

CircRNA-microRNA interactions were anticipated using microRNA predicted estimate software based on TargetScan (Enright et al., 2003) and miRanda (Pasquinelli, 2012) manufactured by Arraystar Corporation. The software analysis procedure is as follows: First, the target estimate was obtained by employing the latest miRNA. Second, using Context +≤ −0.05 and Context ≤ −0.05, candidate miRNAs were concealed. Third, the demanded circRNA-microRNA interactions were acquired following miRNA-related MREs. We used MiRWalk 3.0 to predict the microRNA-related mRNA. The results of differentially expressed mRNAs were reported and overlapped in the gene expression omnibus (GEO) database (GSE 113738). Next, the acquired circRNAs possible target mRNAs’ protein-protein interactions were analyzed to predict the circRNA-regulated hub genes using Cytoscape STRING and molecular complex detection (MCODE) plug-ins.

### Statistical Analysis

Based on our preliminary study, the sample size was chosen to obtain a 0.9 power and *p* < 0.05. Quantitative data are expressed as mean ± standard error (SEM). Data analysis was carried out using GraphPad Prism 6.0. The double-tailed student’s *t*-test was used to compare the statistical significance between groups.

## Results

### Cognitive Dysfunction of Aged Mice on Day 3 Postoperatively

**Figure 1:**
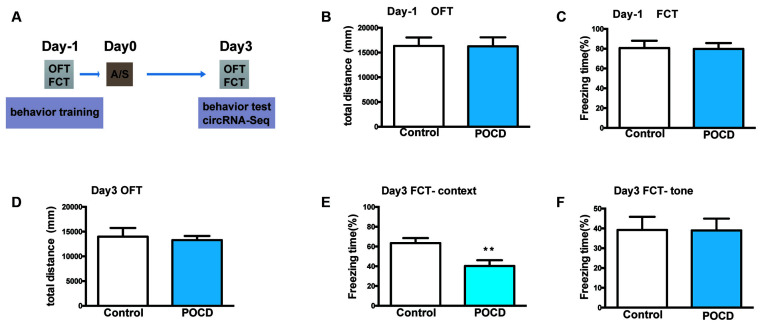
Hippocampus-dependent learning and memory, but not hippocampus-independent learning and memory or motor activity was impaired by anesthesia/surgery in aged mice. **(A)** The experimental process’ schematic diagram. **(B)** The baseline of total distance the elderly mice traveled in open field test (OFT) during the training period 1 day before surgery. **(C)** The baseline of freezing time (%) in fear conditioning test (FCT) of aged mice during the training duration 1 day before surgery. **(D)** The total distance traveled in OFT 3 days after the operation. **(E)** Freezing time percentage in the FCT context test 3 days after surgery. **(F)** Freezing time percentage in the FCT tone test 3 days postoperatively. Data for each group are shown as the mean ± standard error (*n* = 10). **p < 0.01 compared with the control group.

The cognitive function of aged mice was assessed using the OFT and FCT 1 day before and 3 days after the orthopedic surgery under isoflurane anesthesia. The experimental schedule is shown in Figure 1A.

First, the locomotor activity of mice tested by total distance in the OFT suggested that it did not change 1 day before (*p* > 0.05, Figure 1B) or 3 days (*p* > 0.05, Figure 1D) postoperatively. Second, FCT was used to evaluate the learning and memory functions. The context/tone test of the FCT was used to evaluate hippocampus-dependent/independent learning and memory. In the training session, the freezing time of FCT was not significantly changed (*p* > 0.05, Figure 1C), indicating that hippocampus-independent/dependent learning and memory were identical. For the context test, the freezing time was significantly reduced to 3 days postoperatively (*p* < 0.01, Figure 1E). Nevertheless, the freezing time for the tone test was not significantly different (*p* > 0.05, Figure 1F). In summary, the above results indicated that hippocampal-dependent cognition was impaired in aged mice postoperatively, which proves that the POCD mouse model has been successfully established.

### Differentially Expressed circRNAs in POCD of Aged Mice

**Figure 2:**
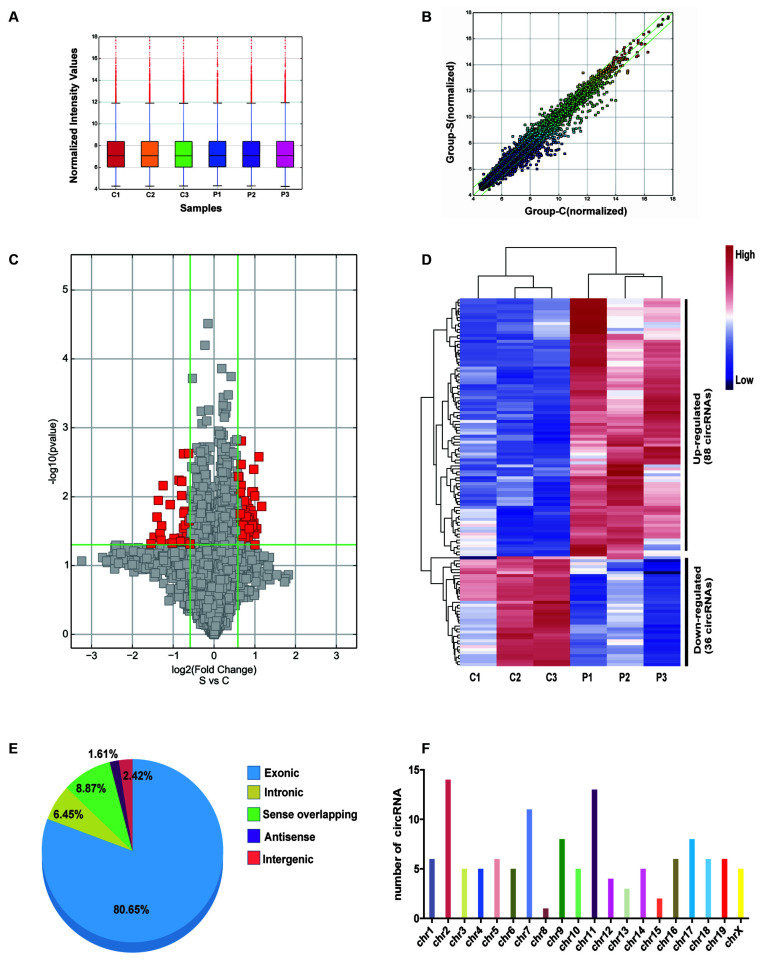
Character of differentially expressed circRNA in the postoperative cognitive dysfunction (POCD) and control groups. **(A)** Distribution of circRNAs in the six samples (C1–3: control group; P1–3: POCD group). **(B)** The difference expression of circRNA is shown by a scatter plot between the control and POCD groups. **(C)** The differential circRNA expression is shown by volcano diagrams between the control and POCD groups. **(D)** Thirty-six downregulated and 88 upregulated circRNAs in POCD are shown in the heatmap. **(E)** The pie chart showed the transcriptional sources of the differentially expressed circRNAs. **(F)** Chromosome locations of the differentially expressed circRNAs.

Differentially expressed circRNAs from the hippocampal tissues of control and POCD mice were screened by microarray-based profiling. The box plot shows a similar data distribution for both groups (Figure 2A). The scatter plot shows the original circRNA expression values between the two groups (Figure 2B). circRNA expression was observed in volcano plots and hierarchical clustering (Figures 2C,D). A total of 124 differentially expressed circRNAs in POCD hippocampal tissues compared with control tissues were identified, of which 88 were upregulated and 36 were downregulated when the fold change was ≥1.5, and the *p*-value was <0.05. The top 10 up/down-regulated circRNAs are listed in Table 2. Additionally, we found that 80.65% of circRNAs were exonic circRNAs, 8.87% were sense-overlapping circRNAs, 6.45% were intronic circRNAs, 2.42% were intergenic circRNAs, and 1.61% were antisense circRNAs (Figure 2E). Localization analysis revealed that these abnormally regulated circRNAs were mainly located in chr1-19 and chrX, but not chr20 and chrY (Figure 2F).

**Table 2:** Top 10 significantly up/downregulated circRNAs in POCD of aged mice.

Significantly up/downregulated circRNAs					
probeID	*P*-value	FDR	FC(abs)	Regulation	circRNA
ASCRP4007024	0.013865474	0.529357952	2.2450955	up	mmu_circRNA_22261
ASCRP4007409	0.00262982	0.529357952	2.1437073	up	mmu_circRNA_27408
ASCRP4003389	0.029190777	0.529357952	2.0674603	up	mmu_circRNA_42249
ASCRP4013349	0.049867064	0.529357952	2.0025764	up	mmu_circRNA_25474
ASCRP4004614	0.034733688	0.529357952	1.9985147	up	mmu_circRNA_44122
ASCRP4010020	0.009783375	0.529357952	1.9958363	up	mmu_circRNA_32165
ASCRP4003327	0.029212904	0.529357952	1.9870759	up	mmu_circRNA_41895
ASCRP4000668	0.024166022	0.529357952	1.9686275	up	mmu_circRNA_22058
ASCRP4002143	0.030499114	0.529357952	1.9626064	up	mmu_circRNA_39251
ASCRP4006538	0.003998226	0.529357952	1.9615141	up	mmu_circRNA_27407
ASCRP4010600	0.048075886	0.529357952	2.9162194	down	mmu_circRNA_33862
ASCRP4001381	0.038744532	0.529357952	2.7699311	down	mmu_circRNA_30227
ASCRP4001879	0.019740651	0.529357952	2.6326581	down	mmu_circRNA_38328
ASCRP4001023	0.011304438	0.529357952	2.5770301	down	mmu_circRNA_22673
ASCRP4010800	0.034227559	0.529357952	2.5008209	down	mmu_circRNA_31065
ASCRP4011284	0.042921723	0.529357952	2.4593752	down	mmu_circRNA_34414
ASCRP4009404	0.02644451	0.529357952	2.4333386	down	mmu_circRNA_45921
ASCRP4011303	0.006882754	0.529357952	2.3732211	down	mmu_circRNA_44559
ASCRP4002514	0.048719463	0.529357952	2.200436	down	mmu_circRNA_29699
ASCRP4002584	0.013002798	0.529357952	2.1034439	down	mmu_circRNA_29984

### Validation of Dysregulated circRNAs Through Quantitative Real-Time Polymerase Chain Reaction (qRT-PCR)

**Figure 3:**
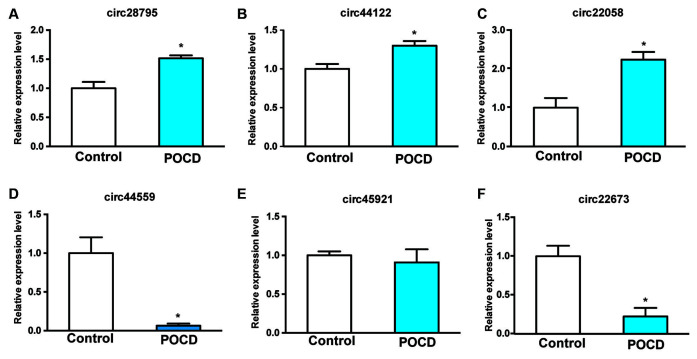
Conformation of the six prospected circRNAs with qRT-PCR. RNAs from the POCD and control groups were involved **(A–F)**. *p < 0.05 compared with the control group.

Six dysregulated circRNAs were chosen randomly, including upregulated (circRNA_28795, circRNA_44122, and circRNA_22058) and downregulated circRNAs (circRNA_44559, circRNA_45921, and circRNA_22673). qRT-PCR demonstrated that circRNA_28795, circRNA_44122, circRNA_22058, circRNA_44559, and circRNA_22673 expression in the control and POCD hippocampal tissues was verified with the microarray results. The expression of circRNA_28795, circRNA_44122, circRNA_22058, circRNA_44559, and circRNA_22673 expression were significantly different (Figures 3A–D,F, *p* < 0.05). The expression of circRNA_45921 was not significantly different (Figure 3E, *p* > 0.05).

### MicroRNA Response Elements (MREs) Analysis

circRNAs may regulate target gene expression through competitive binding with their target miRNAs (Han et al., 2017; Qiu et al., 2018). The circRNA-microRNA networks were predicted using Arraystar’s self-made miRNA target prediction software. A 2D structure was established through sequence analysis of MREs (Supplementary Figures 1A–E). For circRNA_28795, the predicted miRNAs included miR-151-5p, miR-669b-5p, miR-453, miR-138-5p, and miR-298-5p, circRNA_44122, the predicted miRNAs included miR-7033-5p, miR-6914-3p, miR-6974-3p, miR-7010-3p, and miR-361-3p; for circRNA_22058, the predicted miRNAs included miR-196a-5p, miR-7048-3p, miR-322-5p, miR-6964-3p, and miR-670-3p; for circRNA_44559, the predicted miRNAs included miR-1903, miR-207, miR-6946-3p, miR-6896-3p, and miR-6976-3p; for circRNA_22673, the predicted miRNAs included miR-6952-5p, miR-6982-5p, miR-7092-3p, miR-6957-3p, and miR-6986-5p (Table 3).

**Table 3:** Predicted miRNA response elements of the five confirmed circRNAs.

circRNA ID	Predicted miRNA response elements (MREs)				
	MRE1	MRE2	MRE3	MRE4	MRE5
circRNA_28795	miR-151-5p	miR-669b-5p	miR-453	miR-138-5p	miR-298-5p
circRNA_44122	miR-7033-5p	miR-6914-3p	miR-6974-3p	miR-7010-3p	miR-361-3p
circRNA_22058	miR-196a-5p	miR-7048-3p	miR-322-5p	miR-6964-3p	miR-670-3p
circRNA_44559	miR-1903	miR-207	miR-6946-3p	miR-6896-3p	miR-6976-3p
circRNA_22673	miR-6952-5p	miR-6982-5p	miR-7092-3p	miR-6957-3p	miR-6986-5p

### circRNA-miRNA-mRNA Interaction Analysis of Validated circRNAs

**Figure 4:**
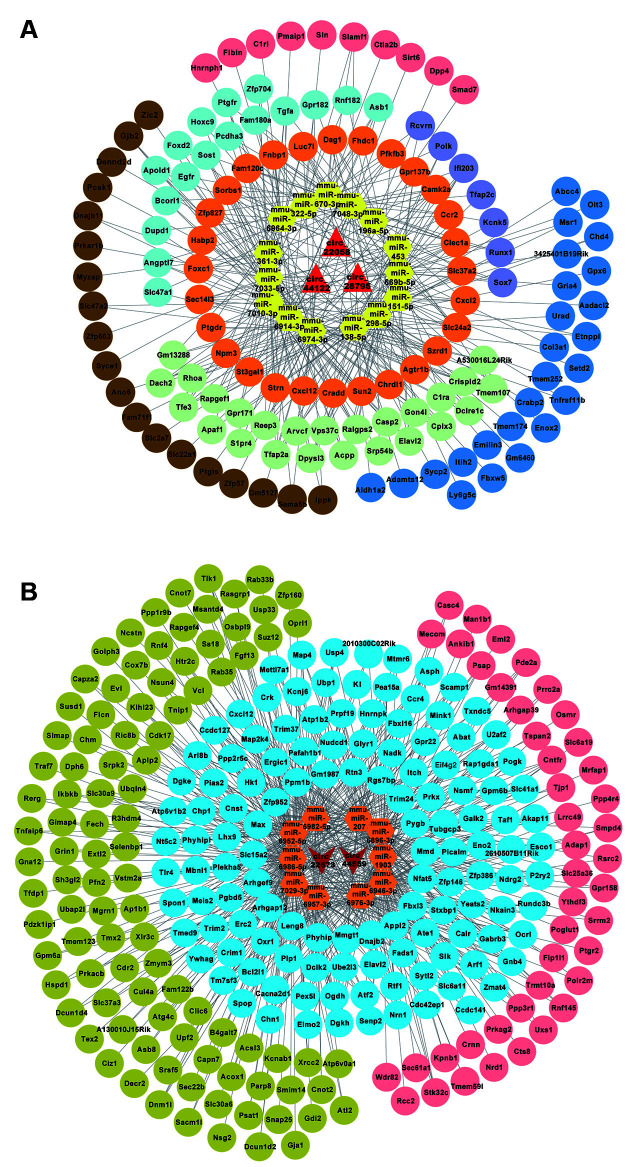
circRNA-miRNA-mRNA network analysis. **(A)** The ceRNA network for the three upregulated circRNAs. **(B)** The ceRNA network for the three downregulated circRNAs.

MiRWalk 3.0 was used to predict target genes of the above-mentioned circRNA-targeted miRNAs. Then, the predicted genes of the three upregulated circRNAs were overlapped by the reported differentially upregulated mRNAs from the GEO database (GSE 113738, from POCD model of aged mice), 131 upregulated mRNAs were screened to build the ceRNA networks, and 268 downregulated mRNAs were screened in the same way. The circRNA-miRNA-mRNA regulatory networks for circRNA_28795, circRNA_44122, and circRNA_22058 included 15 miRNAs and 131 mRNAs (Figure 4A), and for circRNA_44559 and circRNA_22673 included 10 miRNAs and 268 mRNAs (Figure 4B). These results indicate that the ceRNA network might be involved in the POCD mechanism.

### Gene Ontology (GO) and Kyoto Encyclopedia of Genes and Genomes (KEGG) Enrichment Analysis of the Predicted Network Genes

**Figure 5:**
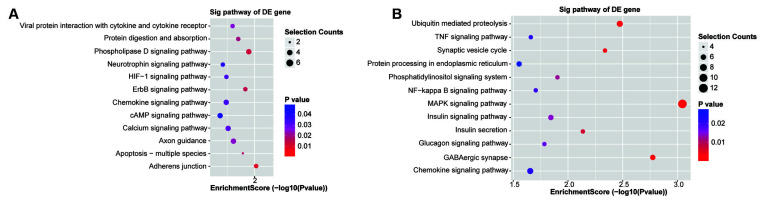
Kyoto Encyclopedia of Genes and Genomes (KEGG) enrichment analysis. **(A,B)** The KEGG enrichment analysis of the up/downregulated circRNA’s predicted genes.

The functions of the 131 upregulated and 268 downregulated genes were analyzed using GO and KEGG analyses. The top highly enriched GO terms of biological process, cellular component, and molecular function for upregulated and downregulated genes are shown in Supplementary Figures 2A,B. The top terms of upregulated genes were identical protein binding (GO:0042802), extracellular region (GO:0005576), and tube morphogenesis (GO:0035239). For downregulated genes, the top terms were protein binding (GO:0005515), cell part (GO:0044464), and localization (GO:0051179). The KEGG pathway analysis revealed that the upregulated genes were involved in the adherens junction, phospholipase D signaling pathway, and ErbB signaling pathway (Figure 5A). Accordingly, the downregulated genes were significantly enriched in the MAPK signaling pathway, GABAergic synapse, and ubiquitin-mediated proteolysis (Figure 5B).

### Protein-Protein Analysis of circRNAs Regulated Genes

**Figure 6:**
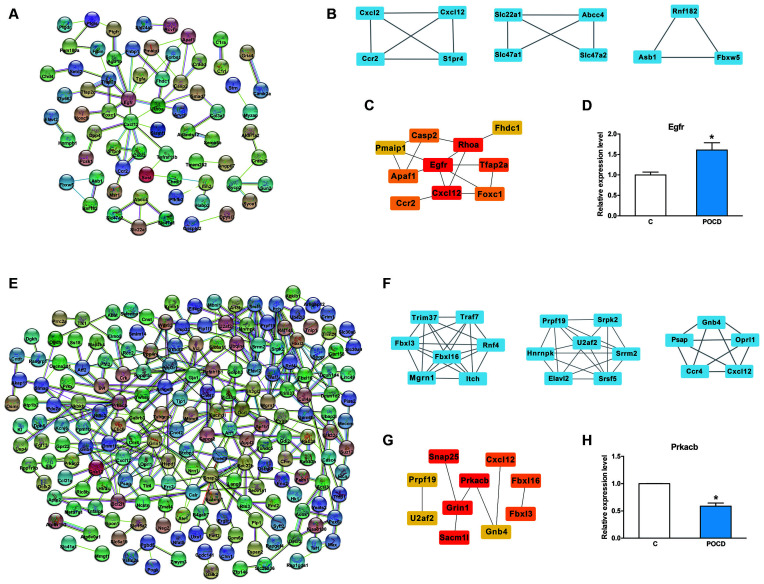
Protein-protein analysis of circRNAs regulated genes. **(A,E)** The protein-protein interaction network analysis for the up/downregulated genes. **(B,F)** Top three sub-network modules that are predicted by MCODE in the Cytoscape software. **(C,G)** Top 10 hub genes screened by CytohHubba in the Cytoscape software. **(D,H)** The mRNA expression level of *Egfr* and *Prkacb* validation. *p < 0.05 compared with the control group.

Further analysis was performed to identify the most promising related genes using the proven circRNAs. Analysis of the protein-protein interaction network for these up/downregulated genes was performed using STRING (11.0 version) under default parameters (Figures 6A,E). Using the Cytoscape plug-in MCODE, the top three sub-network modules were aggregated and extracted from the protein-protein interaction network (Figures 6B,F). For the upregulated network, module A contained four nodes, including cxcl2 and cxcl12. For the downregulated network, module A contained seven nodes, including fbxl3 and fbxl16. Moreover, based on the protein-protein interaction results, the hub genes were screened by degree using CytohHubba of the Cytoscape software. As shown in Figures 6C,G, the hub genes *Egfr/Prkacb* were in the up/downregulated networks, and the mRNA expression levels of *Egfr* and *Prkacb* were also measured by qRT-PCR (Figures 6D,H).

## Discussion

POCD has a high incidence among elderly patients, especially those undergoing major surgeries. The pathogenesis of POCD is complex and lacks effective diagnosis and treatment. Therefore, several recent studies have focused on the epigenetic regulation of the pathogenesis of POCD to discover potential therapies (Li et al., 2015; Liu et al., 2019; Zhong and Xu, 2019). miRNAs and lncRNAs in the brain have been reported to contribute to POCD (Li et al., 2019; Su et al., 2019). However, the roles of circRNAs in POCD remain largely unknown.

CircRNAs are evolutionarily conserved at varying species sequence levels (Jeck et al., 2013). For example, 28% of the mouse circRNA molecules are conserved in humans (Rybak-Wolf et al., 2015). CircRNAs are extremely rich, conserved, and dynamically present in the mammalian brain (Westholm et al., 2014). Because circRNAs are preferentially expressed in nervous genes and nervous tissues (Floris et al., 2017), they are associated with neurological diseases, such as AD, one of the most common neurodegenerative diseases (Burns and Iliffe, 2009). Recent studies have shown that several circRNAs are present in the nervous tissues of patients with sporadic AD and AD mice (Huang et al., 2018; Sekar et al., 2018). Additionally, Cao et al. (2020) reported that dexmedetomidine alleviates POCD through circRNA in aged rats. This study compared the differentially expressed circRNAs between dexmedetomidine and POCD groups; however, our study is the first to examine circRNA profiles between POCD and control groups in the hippocampus of aged mice. Wang et al. (2019) found that circRNA_089763 expression in the plasma exosomes of POCD patients after coronary artery bypass graft surgeries, indicating that circRNA may be a potential biomarker for POCD.

CircRNA has been reported to carry out various kinds of modulating roles, including the interaction with RNA-binding proteins, acting as miRNA sponges, and regulation of paternal gene transcription, mainly at the posttranscriptional and transcriptional levels. In the human genome, a total of 519 canonical miRNA genes have been identified (Denzler et al., 2014; Bartel, 2018). Approximately 70% of the discovered miRNAs are present in the temporary brain and neurons (Cao et al., 2006), implying that miRNAs play a significant regulatory function during the development of the nervous system. As in psychiatric dysfunctions such as schizophrenia (Xu et al., 2013), deregulation of miRNAs is involved in neurodegenerative abnormalities such as Parkinson’s disease (PD) and AD as well (Esteller, 2011). Previous studies have reported a significant correlation that supports the role of miRNAs in POCD (Yu et al., 2015; Wei et al., 2017; Chen et al., 2019; Yazit et al., 2020).

Salmena et al. (2011) proposed the ceRNA hypothesis, suggesting that circRNAs interact with miRNAs to modulate gene expression at the transcriptional or posttranscriptional level. Over the past few years, the ceRNA assumption has been confirmed by many experiments. For instance, circRNA_2837 targets miR-34 family members to regulate LC3-II/p62 and protect neurons by autophagy in the sciatic nerve injury model (Zhou et al., 2018). In neural stem cells, circRNA TTC3 sponges miR-372-3p to regulate TLR4 expression and prevent cerebral ischemia reperfusion injury (Yang et al., 2021). With a new understanding of the ceRNA networks in different diseases, therapies targeting ceRNAs rather than miRNAs alone may become more significant and valuable (Bak and Mikkelsen, 2014). Therefore, GO and KEGG enrichment analyses were carried out for the up/downregulated genes in the ceRNA network. In the GO analyses, the top three terms of the upregulated genes were identical protein binding, extracellular region, and tube morphogenesis; for downregulated genes, the top three terms were protein binding, cell part, and localization. Next, KEGG pathway analysis showed that the upregulated genes included adherens junction, phospholipase D, ErbB signaling pathway, and the downregulated genes involved in the MAPK signaling pathway and mediated proteolysis, which were revealed to be associated with POCD (Li et al., 2014, 2016; Vutskits and Xie, 2016; Zhang et al., 2016; Ding et al., 2017; Lu et al., 2017; Wang D. S. et al., 2018; Wang W. X. et al., 2018; Orser and Wang, 2019; Zheng et al., 2019; Zhong and Xu, 2019). Thus, these bioinformatics analyses of possible pathways also suggested the potential functions of the identified circRNAs in POCD of aged mice.

Meanwhile, based on the protein-protein interaction analysis, *Egfr* and *Prkacb* were predicted in the interaction network. In addition, *Egfr* may be modified by circRNA_22058 and circRNA_44122. *Prkacb* may be modified by circRNA_22673. Egfr has also been proposed as a fundamental disease-associated protein in AD pathogenesis (Quan et al., 2020; Yuen et al., 2020). They have therapeutic abilities through autophagy induction and the attenuation of reactive astrocytes (Tavassoly et al., 2020). *Prkacb* might be involved in the MAPK signaling pathway, GABAergic synapse, insulin secretion, insulin signaling pathway, and chemokine signaling pathway. PKA has also been reported to contribute to neurodegenerative diseases, such as PD and AD (Dagda and Das Banerjee, 2015; Myeku et al., 2016; Kumar and Singh, 2017; Sanders and Rajagopal, 2020; Zhang et al., 2020). PKA has also been reported to be involved in POCD mechanism (Wang W. X. et al., 2018; Zhu et al., 2019). Therefore, the mmu-circRNA_22058 and circRNA_44122/*Egfr* ceRNA network or circRNA_22673/*Prkacb* ceRNA network may contribute to the pathological process of POCD, which will be validated in future studies.

The present study had several limitations. First, we only screened differentially expressed circRNAs in POCD mice 3 days after anesthesia/surgery. Consequently, we did not know whether the differentially expressed circRNAs were time-dependent. Second, although we found that some circRNAs might be associated with POCD, the mechanism needs to be elucidated in further studies.

In summary, we found that circRNAs were differentially expressed in the POCD of aged mice hippocampus. Using bioinformatics analysis of predicted circRNAs, we evaluated the roles and relevant pathways of relative circRNA-target genes.

## Data Availability Statement

The datasets presented in this study can be found in online repositories. The names of the repository/repositories and accession number(s) can be found below: https://www.ncbi.nlm.nih.gov/geo/query/acc.cgi?acc=GSE165798.

## Ethics Statement

The animal study was reviewed and approved by the Animal Care and Use Committee of Xuzhou Medical University.

## Author Contributions

Y-QW and H-HM designed the research. QL, H-BW, CC, HH, and Y-MS contributed to behavioral test and sample collection. L-HM, JW, and Y-YS performed the molecular experiments. Y-QW and H-HM analyzed the bioinformatics data and wrote the article. Y-QW, QL, and H-HM revised the manuscript. All authors contributed to the article and approved the submitted version.

## Conflict of Interest

The authors declare that the research was conducted in the absence of any commercial or financial relationships that could be construed as a potential conflict of interest.

## Publisher’s Note

All claims expressed in this article are solely those of the authors and do not necessarily represent those of their affiliated organizations, or those of the publisher, the editors and the reviewers. Any product that may be evaluated in this article, or claim that may be made by its manufacturer, is not guaranteed or endorsed by the publisher.
