# Supplementary material for: Microarray Analysis Identifies Key Differentially Expressed Circular RNAs in Aged Mice With Postoperative Cognitive Dysfunction
Source: Front Aging Neurosci. 2021 Aug 16;13:716383. doi: 10.3389/fnagi.2021.716383 (PMC8415796; doi:10.3389/fnagi.2021.716383)
Supplement: Supplementary file 1 [file Data_Sheet_1.PDF]

Supplementary figure

Microarray Analysis Identifies Key Differentially Expressed Circular RNAs in Aged Mice with Postoperative Cognitive Dysfunction

Yu-Qing Wu[1], Qiang Liu[1], Hai-Bi Wang[1], Chen Chen[1], Hui Huang[1], Yi-Man Sun[1], Lin-Hui Ma[1], Jie Wan[1], Yin-Ying Sun[1], Hui-Hui Miao[2*]

Supplementary Figure 1

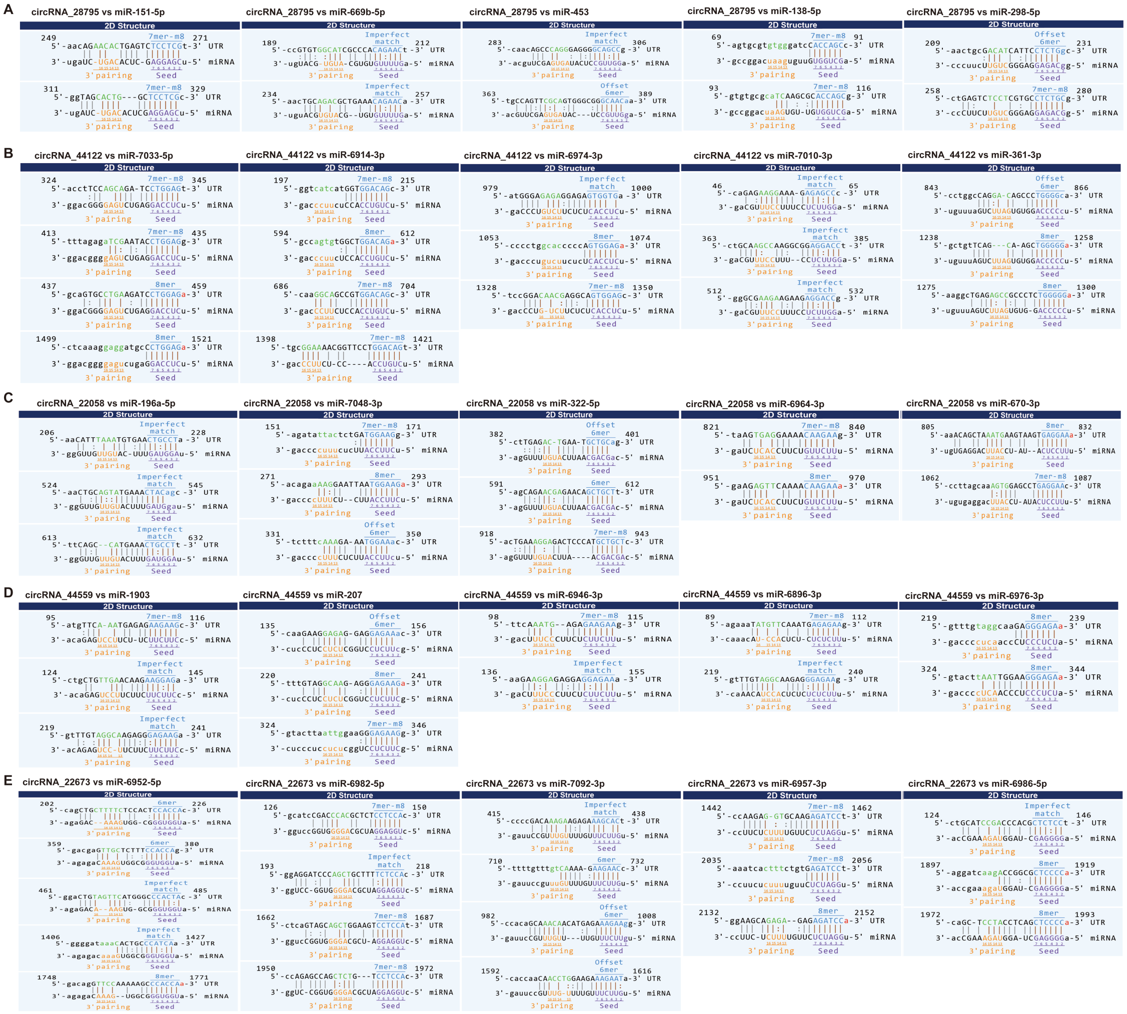

Supplementary Figure 1. The circRNA/miRNA interactions were predicted by Arraystar's self-made miRNA target prediction software based on miRanda and TargetScan. (A-E) Potential miRNA targets of mmu_circRNA_28795 、 mmu_circRNA_44122 、 mmu_circRNA_22058 、 mmu_circRNA_44559 and mmu_circRNA_22673. Local AU: Schematic diagram of the AU weights

on the seed site's both sides. The lack of conserved information about circRNA was indicated by the empty box under "Conservation" between species. M: miRanda; T: TargetScan.

Supplementary Figure 2

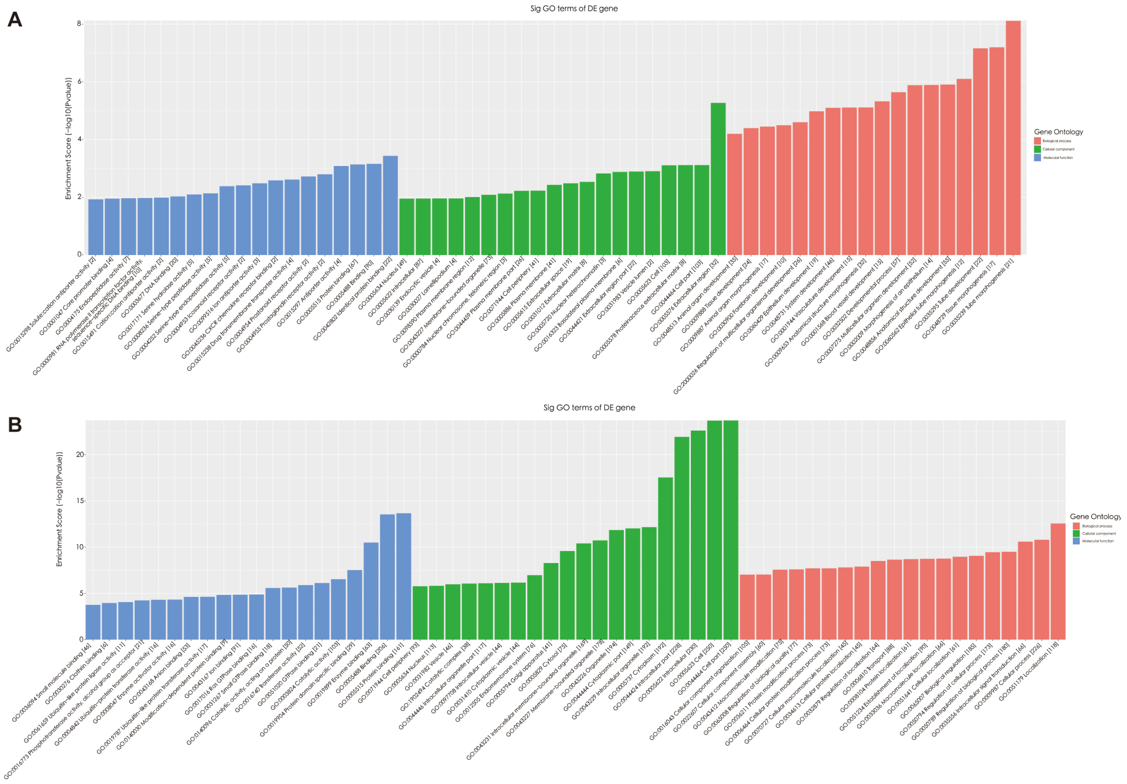

Supplementary Figure 2. GO analysis of the up/down-regulated circRNAs. (A-B) GO enrichment analysis of the up/down-regulated circRNAs that is predicted target genes in terms of BP, CC and MF.
